# Intervention effect of ventilator‑assisted emergency endoscopy in the treatment of cirrhosis‑associated esophagogastric variceal bleeding

**DOI:** 10.20452/wiitm.2024.17908

**Published:** 2024-11-07

**Authors:** Xiu‑Lian Wu, Yanmei Gu, Wenhui Wang, Jiayuan Wei, Xiaoqing Sun, Wenyue Hu, Wenhui He

**Affiliations:** Department of Intensive Care Medicine, Beijing You’an Hospital, Capital Medical University, China

**Keywords:** cirrhosis‑associated esophagogastric variceal bleeding, emergency endoscopy, intervention effect, ventilator

## Abstract

**INTRODUCTION:**

Emergency endoscopy has proven remarkably effective in the treatment of gastrotestinal bleeding. However, its efficacy has not been extensively evaluated specifically in patients with cirrhosis‑associated esophagogastric variceal bleeding (EGVB). The patients may experience stress and anxiety before being subjected to the procedure.

**AIM:**

This study aimed to investigate the effect of ventilator‑assisted emergency endoscopy in the treatment of cirrhosis‑associated EGVB.

**MATERIALS AND METHODS:**

A total of 63 patients with cirrhosis‑associated EGVB were enrolled in the study and divided into 2 groups using the random number table method. The control group (n = 31) received conventional emergency endoscopic hemostasis, while the observational group (n = 32) underwent ventilator‑assisted emergency endoscopic hemostasis. The hemostatic success rate, post‑treatment rebleeding rate, postoperative complication rate, length of stay in the intensive care unit (ICU), cost of hospitalization, and the patients’ feeling of comfort (eg, fever and anxiety) were assessed in both groups.

**RESULTS:**

There were no significant differences in the hemostatic success rate, rebleeding rate, mortality, length of stay in the ICU, or cost of hospitalization between the groups. The symptoms and feelings of anxiety and pain in the observational group were significantly less intense than in the control group. However, there was no significant difference in the frequency of postoperative fever between the groups.

**CONCLUSIONS:**

In the emergency endoscopic treatment of patients with cirrhosis‑associated EGVB, using a ventilator ensures a smooth airway, keeps patients sedated throughout the procedure, and enhances their overall comfort. Ventilator‑assisted emergency endoscopy helps alleviate postoperative pain and reduces anxiety.

## INTRODUCTION 

Hepatitis‑induced cirrhosis oten leads to increased resistance and elevated blood flow in the portal vein, resulting in higher pressure in the hepatic portal venous system.^1^ This causes dilation of the lower esophageal and fundic branches of the stomach and formation varices in the submucosa, which may result it potentially fatal esophagogastric variceal bleeding (EGVB) upon exposure to corrosive acid reflux, increased abdominal pressure, and mechanical injury.^2^ EGVB is a prominent contributor to mortality in patients with cirrhotic portal hypertension (PH).^3^ Studies show that a majority of patients with chronic liver disease who develop cirrhosis are at a risk of gastroesophageal varices. In patients with PH without pre‑existing varices, the annual rate of varices occurrence is 8%, and their incidence closely correlates with liver function. Approximately 40% of patients with Child–Pugh class A cirrhosis have gastroesophageal varices, as compared with 85% of those suffering from Child–Pugh class C cirrhosis.4 EGVB is a severe complication of decompensated cirrhosis, with a mortality rate exceeding 20%.^5^ The condition is characterized by rapid onset and progression, and it poses a serious threat to patients’ lives, as the failure to promptly administer effective hemostatic measures can result in shock or death.^6^^;^^7^^;^^8^^;^^9^

Traditional drug therapy for controlling EGVB has several limitations, including poor efficacy, inability to fundamentally tackle PH caused by cirrhosis, variability in patient response, side effects and potential adverse reactions, as well as drug resistance, which can lead to diminished therapeutic efficacy and the need to continually adjust drug regimens.^10^^;^^11^^;^^12^^;^^13^ In recent years, we have witnessed remarkable advancements in emergency endoscopy and gastroscopy, which have contributed to wider application of these techniques in clinical practice. Of particular interest are the achievements in the management of gastrointestinal bleeding, the benefits of which include precise localization and diagnosis, real‑time treatment and intervention, minimal invasiveness, and multidisciplinary cooperation. Thanks to these advantages, emergency endoscopy and gastroscopy are increasingly preferred in the treatment of gastrointestinal bleeding. These techiniques offer a more convenient, safe, and effective approach to the management of related diseases, and provide strong support for further research.^14^^;^^15^^;^^16^

Upon admission to the hospital, patients with EGVB are often awake. Massive hematemesis or presence of bloody stool is likely to make them nervous and anxious, and endoscopic hemostatic treatment may intensify these feelings by increasing their discomfort and pain. Patient experience is an instrumental determinant in the administration of endoscopic treatment. Endoscopic hemostasis using a ventilator can be performed only after written consent has been obtained from the patient. Therefore, assessing stress and anxiety levels in patients undergoing endoscopic treatment is critical for understanding their choice to proceed with or refuse ventilator‑assisted therapy.^17^^;^^18^ In addition, in all EGVB patients who need and are willing to undergo digestive endoscopy, closely monitored ventilators can be used for sedation / anesthesia and endoscopic treatment.

## AIM 

This study aimed to assess the intervention effect of ventilator‑assisted emergency endoscopy in the treatment of EGVB.

## MATERIALS AND METHODS 

### Clinical data 

A total of 63 patients with EGVB admitted to the intensive care unit (ICU) of the Beijing You’an Hospital, Capital Medical University between May 2021 and January 2023 and treated with emergency endoscopic hemostasis were enrolled in the study.The participants were divided into 2 groups using the random number table method. The control group (n = 31) comprised patients treated with conventional emergency endoscopic hemostasis, while the observational group (n = 32) comprised individuals treated with ventilator‑assisted emergency endoscopic hemostasis. The inclusion criteria were 1) presence of decompensated cirrhosis and EGVB; 2) lack of cognitive impairment and ability to cooperate before the treatment; 3) presence of hematemesis, hematochezia, or both; and 4) treatment with emergency endoscopy. We excluded patients with noncirrhosis‑associated EGVB, those suffering from a loss of consciousness, hepatic encephalopathy, or shock who were unable to cooperate before the treatment, and individuals with other chronic conditions, such as cardiovascular and cerebrovascular diseases and diabetes mellitus.

### Treatment methods 

All endoscopic procedures were performed by board‑certified gastroenterologists with a minimum 5‑year experience in emergency endoscopy. Each operating physician had also completed specialized training in advanced endoscopic techniques, ensuring a high level of expertise and consistency in performing the procedures. During the treatment, no specific medications were administered for anxiety. For pain management, subcutaneous injections of morphine, intramuscular injections of tramadol and promethazine, or intravenous infusions of propofol and morphine with midazolam were generally used.

### Preoperative preparation 

All patients received standard care, including electrocardiographic monitoring, invasive blood pressure monitoring, and the preparation of first‑aid drugs and resuscitation supplies. Massive bleeding shock was managed promptly. Blood, biochemical, blood typing, and coagulation function tests were conducted, and blood matching was completed before the emergency endoscopic treatment. The patients were fasted, given oxygen inhalation, and informed about the purposes and procedures of the study. Intravenous access was also established, and deep venous catheters and urinary catheters were placed. Hemostatic and acid‑suppressing drugs were administered to ensure circulatory stability.

### Control group procedure 

The patients in the control group were placed in the left lateral position for gastroscopy and treated with a sclerosing agent, endoscopic variceal ligation (EVL), or drug surface spraying, according to their endoscopy results. If endoscopy revealed the presence of a vascular lesion or aneurysm, a sclerosing agent was injected to induce hardening and occlusion of the blood vessels at the lesion. In the case of bleeding points or smaller vascular lesions, EVL was employed. If surface ulcers or mucosal lesions were found on endoscopy, drug surface spraying was applied to facilitate healing and control the bleeding. A Sengstaken–Blakemore tube was placed preoperatively for intraoperative bleeding control via compression and was removed after the procedure, leaving no further gastric tube for gastric fluid drainage.

### Observational group procedure 

The patients in the observational group were intubated in the anesthesiology department while awake. Once the endotracheal tube was fixed and moved to the right corner of the mouth, the patients were placed in the left lateral position for endoscopy under intubation and were treated with sclerosing agents, EVL, or drug surface spraying, according to their endoscopy results. A Sengstaken–Blakemore tube was placed preoperatively for intraoperative bleeding control via compression and was removed postoperatively, leaving no further gastric tube for gastric fluid drainage. The patients were placed on a ventilator for a total of 5 to 23 hours and extubated after waking up.

### Observational indices 

During hospitalization, the following parameters were recorded: body mass index (BMI; calculated as weight in kilograms divided by height in meters squared); course of the disease (defined as progression from the onset to recovery or deterioration), etiology of cirrhosis (posthepatitis cirrhosis, alcoholic cirrhosis, or a combination thereof), bleeding site (the gastric fundus or both the esophagus and gastric fundus), and severity of cirrhosis (compensatory or decompensatory stage). Additionally, all patients were assessed after ICU discharge and several measures were collected: 1) hemostatic success rate, defined as effective control of bleeding symptoms during a specific procedure, where the bleeding is stopped without reoccurring for a period of 72 hours following the treatment. Successful hemostasis was determined once the patient stopped vomiting blood after the treatment, their stool gradually turned from tarry or bloody to normal, their vital signs became stable, and there was no further decline in their hemoglobin level^19^; 2) rebleeding rate, that is, recurrence of hematemesis or melena following gastroscopic hemostatic treatment. Rebleeding was confirmed by a further decrease in hemoglobin levels following blood transfusion, necessitating emergency compression hemostasis with a 3‑lumen, 2‑balloon tube or a second endoscopic hemostatic treatment; 3) mortality rate, defined as the number of deaths due to EGVB during the period from the completion of the hemostatic treatment to stabilization of the patient’s condition and their discharge from the ICU; 4) post‑treatment patient comfort, which included postoperative stomach pain, burning sensation, or fever. Pain was considered an inevitable sensory response to tissue damage.^20^ Patient‑related data were collected using the Numerical Rating Scale (NRS), a widely used assessment tool that expresses the degree of pain numerically. Typically, the scale requires patients to pick a number to describe their current pain level, where 0 corresponds to no pain and 10 indicates the most severe pain. Patients select the appropriate number based on their personal sensations to provide a relatively objective assessment of the experienced pain. Anxiety was defined as the feeling of fear that occurs when faced with threatening or stressful situations.^21^ The Hamilton Anxiety Scale was utilized to assess patient anxiety; and 5) the length and cost of stay in the ICU.

### Ethics 

This study was approved by the Ethical Approval Committee of Beijing Youan Hospital (JYKLY[2023]337), and all patients or their families provided written informed consent to participate. This study was conducted in accordance with the Declaration of Helsinki.

### Statistical analysis

Detailed statistical analysis of the data collected in this study was performed using the SPSS 19.0 statistical software. Numerical variables were described using mean (SD), and the t test was used for intergroup comparisons. Categorical variables were presented as the number of cases and percentages, and the χ^2^ test was used for intergroup comparisons. Statistical significance was established at P below 0.05.

### RESULTS 

### General clinical data 

The study population comprised 51 men and 12 women, and the age range was 34 to 67 years. A total of 31 patients presented with posthepatitis cirrhosis, 19 had alcoholic cirrhosis, and 13 suffered from a combination of both. Nine individuals required transition from Sengstaken–Blakemore tube compression hemostasis to emergency endoscopic hemostasis. In the control group there were 27 men (87.1%) and 4 women (12.9%) at a mean (SD) age of 56.84 (14.81) years. The observational group included 24 men (75%) and 8 women (25%) at a mean (SD) age of 52.38 (16.71) years. No significant differences were found between the groups with respect to sex and age distribution. In addition, there were no significant differences in terms of the etiology of cirrhosis, bleeding site, BMI, course of the disease, degree of cirrhosis, and preoperative hemodynamic parameters or laboratory test results. A comparison of baseline clinical characteristics is presented in TABLE 1.

### Hemostasis effect, rebleeding rate, and mortality

There were no significant differences in the hemostatic success rate (87.09% vs 87.5%), rebleeding rate (6.45% vs 9.37%), or mortality (6.45% vs 3.12%) between the control and observational groups. Details are illustrated in TABLE 2 .

### Length of stay and hospitalization expenses 

There were no significant intergroup differences with respect the length of stay in the ICU (mean [SD], 4.16 [0.93] days vs 6.48 [1.02] days, respectively, for the control and observational groups) or the cost of hospitalization (mean [SD], 47300 [9400] RMB vs 66 800 [8800] RMB, respectively, for the control and observational groups). The additional costs associated with ventilator use and related disposable consumables in the observational group did not result in a significant difference in the overall cost of ICU hospitalization between the 2 groups.

**TABLE 1:** Comparison of general patient data

Parameter		Control group (n = 31)	Observational group (n = 32)	*P *value
Sex	Men	27	24	0.22
	Women	4	8	
Age, y		56.84 (14.81)	52.38 (16.71)	0.06
BMI, kg/m2		21.37 (4.38)	22.69 (4.76)	0.57
Disease duration, y		2.84 (1.73)	2.76 (1.59)	0.44
Etiology of cirrhosis	Posthepatitis cirrhosis	18	13	0.34
	Alcoholic cirrhosis	7	12	
	Both	6	7	
Bleeding site	Fundus of the stomach	24	19	0.12
	Esophagus and gastric fundus	7	13	
Severity of cirrhosis	Compensatory period	22	19	0.34
	Decompensation period	9	13	

Data are presented as number of patients or mean (SD). Abbreviations: BMI, body mass index

**TABLE 2:** Comparison of hemostasis effect, rebleeding rate, and mortality

Group	Hemostasis success	Rebleeding	Mortality
Control group (n = 31)	27 (87.09)	2 (6.45)	2 (6.45)
Observational group (n = 32)	28 (87.5)	3 (9.37)	1 (3.12)
*P *value	>0.99	>0.99	0.61

Data are presented as number (percentage) of patients.

### Post-treatment comfort

The incidence of post‑operative pain was lower in the observational group than in the control group (8 [25%] vs 22 [70.96%]; P <0.001), and anxiety was also less prevalent among the patients treated with ventilator‑assisted endoscopy, as compared with those treated with the conventional approach (9 [28.12%] vs 30 [96.77%]; P <0.001). However,

the difference in the incidence of postoperative fever between the groups was not significant (7 [21.87%] vs 13 [41.93%], respectively, in the observational group vs the control group)TABLE 3 .

## DISCUSSION 

EGVB is an urgent and critical condition, and emergency endoscopic hemostasis remains the mainstay of its treatment. Given its characteristics, including acute onset, rapid progression, and massive bleeding, it is critical to identify the bleeding site (via endoscopy), assess its severity, and perform hemostasis quickly and accurately. However, in the acute stage of upper gastrointestinal hemorrhage, heavy bleeding can obscure the operative field and impact the patient’s condition, possibly complicating or even impeding emergency endoscopy.^22^^;^^23^^;^^24^^;^^25^^;^^26^ A previous study revealed a reduced length of stay and lower hospitalization expenses for endoscopy performed within 8 hours of the bleeding onset, highlighting the significant economic value of early endoscopy.^27^ Notably, ventilator‑assisted therapy did not increase medical expenses. Emergency endoscopic procedures can induce a state of anxiety in patients and lead to pronounced reactive sympathetic excitation, triggering multiple organ responses and severely affecting disease prognosis.^28^ This study investigated the effects of ventilator‑assisted endoscopy on treatment results, hospitalization costs, and patient comfort by comparing the outcomes of patients undergoing emergency endoscopy assisted by tracheal intubation with the outcomes of those undergoing conventional emergency endoscopy. The results suggest that ventilator‑assisted emergency endoscopy does not significantly affect treatment efficacy or hospitalization duration, but it does increase hospital costs due to the additional use of a ventilator. The patients remain sedated throughout the procedure, which is associated with reduced discomfort. Tracheal intubation can prevent severe hematemesis that could result in aspiration. While ventilator‑assisted therapy can shorten the length of ICU stay, this finding was not statistically significant.^29^^;^^30^ Patients receiving ventilator‑assisted therapy experienced less stomach pain, burning, and anxiety during and after the procedure, as compared with those treated with conventional endoscopy.^31^ However, a larger sample size is needed to further validate the study results. The additional expenses associated with ventilator‑assisted therapy did not significantly affect the overall cost of care. This suggests that additional expenses incurred by certain aspects of treatment can be balanced in other cost‑saving ways, making ventilator‑assisted therapy economically viable. The type of sedation used depends on the patient’s age, medical history, and other factors.^32^ Numerous sedatives have been validated for digestive endoscopy, including midazolam, propofol, fentanyl, and meperidine. However, there are many concerns related to their use in such procedures, including aderse events, sedation considerations for different populations, and legal issues.^33^ Aksu^34^ found that listening to music before the examination reduced stress and anxiety levels in patients undergoing gastrointestinal endoscopy and improved its success rate.

**TABLE 3:** Comparison of pain, anxiety, and postoperative fever occurrence

Group	Pain	Anxiety	Fever
Control group (n = 31)	22 (70.96)	30 (96.77)	13 (41.93)
Observational group (n = 32)	8 (25)	9 (28.12)	7 (21.87)
*P *value	<0.001	<0.001	0.11

Data are presented as number (percentage) of patients.

Certain limitations of the study need to be acknowledged. First, the relatively small sample size limits the generalizability of its findings. What is more, the specific characterictics of the study population may prevent the findings from being applied to other patient groups with upper gastrointestinal hemorrhage. To address these limitations in future research, the sample size should be increased to include more cases, enabling a more comprehensive assessment of the effects of ventilator‑assisted therapy. In addition, a more comprehensive psychological assessment should be performed to evaluate patient comfort.

## CONCLUSIONS 

In summary, this study preliminarily revealed that the use of a ventilator ensures a smooth airway, keeps patients sedated, and enhances their overall comfort during emergency endoscopic treatment of cirrhosis‑associated EGVB. Ventilator‑assisted emergency endoscopy helps alleviate postoperative pain and reduces anxiety. Therefore, it is an effective treatment method for patients with cirrhosis‑associated EGVB and merits clinical application. Despite specific economic factors, ventilator‑assisted emergency endoscopy does not significantly increase overall medical costs. Our findings provide a useful reference for the practical application of this therapeutic modality; however, further studies are needed to verify and refine this conclusion. Such studies should focus on increasing the sample size, including a more heterogenous patient group, and evaluating psychological status

of the patients to assess the value of ventilator‑assisted therapy more comprehensively in varying contexts.
